# In Search of Biomarkers for Pathogenesis and Control of Leishmaniasis by Global Analyses of *Leishmania*-Infected Macrophages

**DOI:** 10.3389/fcimb.2018.00326

**Published:** 2018-09-19

**Authors:** Patricia Sampaio Tavares Veras, Pablo Ivan Pereira Ramos, Juliana Perrone Bezerra de Menezes

**Affiliations:** ^1^Laboratory of Host-Parasite Interaction and Epidemiology, Gonçalo Moniz Institute, Fiocruz-Bahia, Salvador, Brazil; ^2^National Institute of Tropical Disease, Brasilia, Brazil; ^3^Center for Data and Knowledge Integration for Health, Gonçalo Moniz Institute, Fiocruz-Bahia, Salvador, Brazil

**Keywords:** biomarkers, leishmaniasis, macrophages, RNA-seq, proteomics, global analysis, functional enrichment analysis

## Abstract

Leishmaniasis is a vector-borne, neglected tropical disease with a worldwide distribution that can present in a variety of clinical forms, depending on the parasite species and host genetic background. The pathogenesis of this disease remains far from being elucidated because the involvement of a complex immune response orchestrated by host cells significantly affects the clinical outcome. Among these cells, macrophages are the main host cells, produce cytokines and chemokines, thereby triggering events that contribute to the mediation of the host immune response and, subsequently, to the establishment of infection or, alternatively, disease control. There has been relatively limited commercial interest in developing new pharmaceutical compounds to treat leishmaniasis. Moreover, advances in the understanding of the underlying biology of *Leishmania* spp. have not translated into the development of effective new chemotherapeutic compounds. As a result, biomarkers as surrogate disease endpoints present several potential advantages to be used in the identification of targets capable of facilitating therapeutic interventions considered to ameliorate disease outcome. More recently, large-scale genomic and proteomic analyses have allowed the identification and characterization of the pathways involved in the infection process in both parasites and the host, and these analyses have been shown to be more effective than studying individual molecules to elucidate disease pathogenesis. RNA-seq and proteomics are large-scale approaches that characterize genes or proteins in a given cell line, tissue, or organism to provide a global and more integrated view of the myriad biological processes that occur within a cell than focusing on an individual gene or protein. Bioinformatics provides us with the means to computationally analyze and integrate the large volumes of data generated by high-throughput sequencing approaches. The integration of genomic expression and proteomic data offers a rich multi-dimensional analysis, despite the inherent technical and statistical challenges. We propose that these types of global analyses facilitate the identification, among a large number of genes and proteins, those that hold potential as biomarkers. The present review focuses on large-scale studies that have identified and evaluated relevant biomarkers in macrophages in response to *Leishmania* infection.

## Introduction

Leishmaniasis is a neglected parasitic disease that is distributed worldwide and is often associated with poverty. Most cases of this disease arise in developing countries and result in 20,000–40,000 deaths per year. *Leishmania*, the causative agent, is transmitted to vertebrate hosts, including humans, by a bite from the sand fly during blood-feeding. Its pathogenesis involves the stimulation of different types of host immune responses that result in distinct clinical outcomes (Scorza et al., 2017), including cutaneous, mucocutaneous, and visceral manifestations, depending on the parasite species and host genetic background (Bañuls et al., 2011). Localized or mucocutaneous forms of tegumentary leishmaniasis, e.g., those caused by *Leishmania braziliensis*, induce activation of the host immune response, resulting in an immune-mediated pathology that manifests as localized ulcerations in human skin or disfigurement involving the nasal and oropharyngeal mucosa (Gupta et al., 2013). By contrast, the visceral form of this disease arises from parasites of the *L. donovani* complex and may result in severe systemic manifestations and high morbidity and mortality due to the inhibition of host inflammation and immunity (Das et al., 2014).

Neutrophils, dendritic cells, and macrophages, the main host cells that harbor parasites, are immune cells that are recruited to the infection site, where they recognize parasites that, once internalized, multiply within their phagolysosomes. In addition, these cells produce cytokines and chemokines that contribute to lymphocyte recruitment, which is critical to the disease outcome (Liu and Uzonna, 2012).

Measures designed to eradicate leishmaniasis necessitate a combination of intervention strategies, including early diagnosis and treatment. In visceral leishmaniasis, diagnostic procedures both evaluate clinical signs and employ parasitological or serological testing that is potentially capable of discriminating active visceral leishmaniasis from its asymptomatic form. By contrast, clinical evaluations are of greater importance in cutaneous and mucocutaneous leishmaniasis because serological testing is inadequate. Since leishmaniasis treatment must be affordable to ensure access by affected impoverished populations, the development of new compounds to treat leishmaniasis has attracted limited commercial interest. In addition, studies unveiling several aspects of the host response to *Leishmania* infection have not resulted in the discovery of effective new therapeutic interventions. Although some new alternative anti-leishmanials have recently emerged, none are considered ideal due to their high toxicity, prolonged treatment duration, and severe adverse reactions, which can lead to treatment abandonment and frequent cases of relapse (Aronson et al., 2017).

Biomarkers as surrogate endpoints have been recommended for use in clinical trials to aid in the early diagnosis of leishmaniasis since primary clinical markers are sparse and are only applicable after an extensive follow-up period. Biomarkers offer another advantage in that they allow measurements to be obtained more rapidly and in a less invasive context than do conventional clinical or parasitological evaluations. They could also facilitate the design of smaller, more efficient clinical studies that may lead to expedited regulatory evaluation and treatment approval. A recent elegant systematic review identified different types of direct and indirect biomarkers that were shown to be involved in *Leishmania* infection and disease outcome. Among the 170 studies evaluated, 53 potential pharmacodynamic biomarkers were identified, including direct, i.e., of parasite origin, and indirect, i.e., from host cells, markers of cutaneous, post-kala-azar dermal leishmaniasis, and visceral leishmaniasis (Kip et al., 2015).

The identification of a set of soluble biomarkers in host tissue has been exploited using sera of individuals with visceral leishmaniasis (Solcà et al., 2016; Araújo-Santos et al., 2017). A recent study screened a variety of soluble molecules and identified a set of inflammatory biomarkers that grouped together under a hierarchical cluster analysis (Araújo-Santos et al., 2017). A significant increase in the levels of the following inflammatory mediators was observed: resolvin D1 (RvD1), leukotriene B4 (LTB4), prostaglandin F2α (PGF2α), IL-1β, IL-6, IL-8, IL-10, IL-12p70, and TNF-α, in contrast to a decrease in TGF-β1 in the serum of patients with visceral leishmaniasis compared with an uninfected endemic control group. After 30 days of therapy, the authors observed that individuals clustered together in terms of decreases in the levels of these inflammatory molecules, distinct from the individuals with active infection, thus reinforcing the idea that this set of soluble molecules might function as biomarkers for the host response to therapy. These authors further remarked that the modulation observed in the concentrations of these markers provides evidence that “an inflammatory imbalance hallmarks active visceral leishmaniasis disease,” which more importantly can greatly aid in the design of new interventions (Araújo-Santos et al., 2017). Another recent study focused on the identification of circulating biomarkers of “inflammation, immune activation, oxidative stress, and anti-sand fly saliva IgG concentrations” in canine sera to characterize biosignatures associated with the severity of visceral leishmaniasis in dogs presenting a variety of clinical manifestations (Solcà et al., 2016). These authors discovered unique biosignatures according to the frequency and intensity of clinical signs. A characteristic signature was found to be associated with animals presenting severe visceral leishmaniasis, as evidenced by a gradual decrease in LTB_4_ and PGE_2_ concomitant with a gradual increase in CXCL1 and CCL2. Furthermore, the quantification of three mediators, LTB_4_, PGE, and CXCL1, was shown to correlate with different clinical scores. This study clarified that visceral leishmaniasis severity in dogs can be associated with inflammatory profiles, which are distinguishable according to clinical presentation, via the expression of circulating eicosanoids and chemokines.

Advances in global genomic and proteomic analysis techniques have enabled the identification and characterization of pathways involved in the infection process in both parasites and the host. These approaches have been lauded due to their greater effectiveness than focusing exclusively on individual molecules, which rarely lend insight into disease pathogenesis. RNA-seq and proteomics, both large-scale techniques designed to characterize genes or proteins in a given cell line, tissue, or organism, offer the advantage of a more global and integrated view of the myriad biological processes that occur within cells (Wang et al., 2009; Veras and Bezerra De Menezes, 2016).

The analytical tools that are available for studying complex data include functional enrichment analysis (e.g., the widely adopted GSEA), in which a set of transcriptionally disturbed genes belonging to a common group of canonical pathways or biological processes reflect alterations in the pathways themselves. Gene co-expression networks can also be used to infer which genes are related to an infectious process. These networks offer the distinct advantage of enabling the discovery of previously unknown relationships by building on the notion of “guilty by association” (Huang da et al., 2009a; Kuleshov et al., 2016). A significant advantage of integrating genomic and proteomic information is that these data can be used in rich multi-dimensional analyses that allow identification from an enormous pool of expressed genes and proteins those that offer promise for use as biomarkers of different endpoints in leishmaniasis, such as disease diagnosis and treatment, in addition to markers for disease establishment and progression.

It has been clearly shown that macrophages are not only the major cells that harbor *Leishmania* parasites, but are also those that modulate host immune response by producing cytokines and presenting parasite antigens to T cells (Podinovskaia and Descoteaux, 2015). In addition, it has been shown that initial interactions between *Leishmania* parasites and macrophages contribute to the outcome of infection (Laskay et al., 1995; Scharton-Kersten and Scott, 1995). Thus, the present review focuses on recent large-scale studies detailing the host-related genes analyzed by RNA-seq and the proteins identified by proteomics, as well as describes the types of bioinformatics analyses used to integrate the large volumes of data generated by these high-throughput sequencing techniques. Due to the importance of these cells in the host response to *Leishmania* infection, we endeavor to review those genes and proteins expressed by macrophages in response to infection by this parasite that offer potential as future targets for use as indirect markers of pathogenesis or as targets for therapeutic intervention.

## Decoding data into knowledge: bioinformatic strategies to analyze, integrate, and interpret high-throughput *omics* data

Over the last decade, the biomedical field has witnessed a tremendous increase in its capabilities to generate data. With large initiatives such as the 1000 Genomes Project (1000 Genomes Project Consortium et al., 2015) (which expanded upon the foundation established by the Human Genome Project), ENCODE (Encode Consortium, 2012), the Genotype-Tissue-Expression (GTEx) Project (GTEx Consortium, 2013), among others, the performance of large-scale *omics* investigations has gained more traction, and the adoption of high-throughput technologies is now widespread. The development of novel analytical strategies to decode and transform these data into knowledge is of paramount importance. In this section, we begin by reviewing traditional bioinformatics tools that can be applied to the analysis of high-throughput datasets. Next, we present more recent, complementary approaches that have yet to become entirely embraced by the community, paralleled by the development of computational techniques used by the scientific community working with leishmaniasis.

### Differentially expressed molecules: only the tip of the iceberg

Traditional RNA-seq analyses begin by identifying genes with significantly altered expression across groups of samples, yielding a list of differentially expressed genes (DEGs). Statistical strategies for detecting DEGs based on RNA-seq-derived count data rely mainly on the use of Poisson or negative binomial distributions. The first has the advantage of being simpler, with a single parameter, λ, entirely defined by the mean, and having a variance equal to the mean. However, this property limits its application when biological replicates are available, when this assumption regarding variance does not hold because biological replicates typically present high variability (Bullard et al., 2010). In contrast, the negative binomial distribution, specified by the mean μ and variance σ^2^, is considered a more appropriate alternative since its variance is always greater than or equal to the mean. It also allows modeling of the mean-variance relationship typically observed in RNA-seq count data (Oberg et al., 2012). Computational tools that use the negative binomial include edgeR (Robinson et al., 2010), DESeq2 (Love et al., 2014), and baySeq (Hardcastle and Kelly, 2010), among others. Limitations of the negative binomial distribution include the observation that, in practice, μ and σ^2^ are usually estimated from the data, which can be problematic when only a few replicates are available, as is still common practice for many high-throughput experiments, given budget constraints. These methods also suffer when the distributional assumptions in the input data do not hold, and non-parametric strategies have been proposed as more reliable alternatives in these cases (Tarazona et al., 2011; Li and Tibshirani, 2013). Other strategies, such as data transformation using *voom* (Law et al., 2014), in which the mean-variance trend is modeled in a non-parametric fashion, allow the subsequent use of traditional microarray packages, e.g., *limma* (based on normal distribution assumptions) and other microarray-specific downstream analyses. Many studies have compared the performance of these algorithms under various scenarios, including the variation in the number of replicates, sequencing depth, and the use of other tools concomitantly, such as in mapping steps (Rapaport et al., 2013; Soneson and Delorenzi, 2013; Law et al., 2014; Zhang et al., 2014; Schurch et al., 2016; Costa-Silva et al., 2017; Sahraeian et al., 2017; Williams et al., 2017). The wealth of methods available is indicative that there is no “one-tool-fits-all” approach for detecting DEGs in RNA-seq data and suggests that the *a priori* delineation of the experimental design together with knowledge of the biological question addressed is crucial in choosing the best set of tools and parameters.

A list of DEGs, however, is only a first step toward defining the biological processes that appear altered in a given experiment, and in most settings, the sole study of these genes will be too reductionist in nature and mostly ineffective as it must be conducted in a gene-by-gene fashion. Additionally, an intrinsic problem when detecting DEGs is the need to establish thresholds for *p*-values (or multiple testing corrected *p*-values) and fold-changes (FC), which can be largely arbitrary and lead to the loss of true DEGs (if too conservative) or their false inclusion (if too relaxed). The finding that the different DEG tools present slightly different true positive and false positive rates performance further complicates the matter (Schurch et al., 2016). To illustrate this point, recently published studies in the leishmaniasis field such as that of Christensen et al. (2016) for instance, used cut-offs of absolute FC ≥ 2 and a Benjamini-Hochberg corrected *p*-value ≤ 0.05 to call DEGs, as did Masoudzadeh et al. (2017). Others, such as Kong et al. (2017) have relied on a consensus strategy among different approaches, where a gene was considered differentially expressed if three methods positively identified it as a DEG, and gene lists with varying strictness for the corrected *p*-values (< 0.001 and < 0.01) were generated. To circumvent some of these issues and obtain a more expanded view of *omics* datasets, other analytical approaches can be used, and we detail some alternatives in the two sections that follow.

Proteomics allows the identification and quantification of many (usually thousands) of proteins present in a given sample. Recent advances in the experimental approaches available for accessing the proteome have allowed an improved resolution, with less input material, when compared to more classical techniques such as 2D gel electrophoresis (2D-GE) that can be followed by liquid chromatography coupled to mass spectrometry (LC-MS). Proteome quantification using MS can be generally classified as label-based or label-free approaches. The first relies on the differential labeling of samples using stable isotopes (such as ^2^H, ^13^C, ^15^N, and ^18^O) followed by quantification using MS. Technological improvements in the field of MS and chromatography have leveraged the development of high-throughput proteomic analyses that permit a higher proteome coverage and are collectively termed label-free quantitative proteomics (LFQP), a highly accurate method that presents less susceptibility to technical errors. LFQP relies on measurements of individual samples by MS, and quantifies proteins based on either peak intensity or spectral counts of each peptide. Each of these broad techniques have their specificities regarding sample preparation, purification, separation, and ionization method, making the recommendation of specific computational tools for their analysis particularly more challenging than for RNA-seq data. For instance, the choice of labeling method will inform the corresponding choice of appropriate analytical packages, and a software that works well for ^15^N label-based quantification may not be suitable for analyzing ^18^O data (Anand et al., 2017). For this reason, we refer the reader to in-depth reviews that have tackled the methodological aspects related to the analysis of raw proteomic data (Mueller et al., 2008; Haga and Wu, 2014; Sandin et al., 2014; Kuharev et al., 2015; Navarro et al., 2016; Ramus et al., 2016; Välikangas et al., 2017), while focusing, for this review, on computational tools that use pre-processed data as input for downstream analyses. Albeit different in nature, proteomic studies suffer from similar concerns as those raised for RNA-seq data, in that the sole examination of a list of differentially expressed proteins across conditions (also constructed using *ad-hoc* criteria) may lead to loss of important biological aspects of the data. Rather, we argue that those working with high-throughput *omics* datasets will benefit from more ample analyses, such as those discussed in the following sections.

### Enrichment analyses allow a contextualization of altered biological processes in high-throughput data

While the identification of expression changes at the gene-level allows one to conveniently explain and validate small phenomena, such as by quantitative RT-PCR assays, the use of integrative approaches permits a broader understanding of the biological processes that underlie more complex questions, such as infection of the host cell by a pathogen. The contextualization of genes into pathways and other more general cellular processes effectively reduce the need to interpret causation at the gene-level and simultaneously reduce the dimensionality of the problem, as a single pathway is usually composed of several genes that act in concert to perform their cellular function.

Enrichment analysis tests whether, for a given set of events of interest (that could be DEGs or proteins, or groups of co-expressed molecules), there is over-representation (*enrichment*) of associated biological features than would be expected by chance. These biological features are usually cellular processes based on a common vocabulary (or ontology), including the Gene Ontology (GO) (The Gene Ontology Consortium, 2017), KEGG, and Reactome pathways (Kanehisa et al., 2017; Fabregat et al., 2018), as well as other more specific biological states such as oncogenic- and immunological-related ones, e.g., those from the MSigDB, which also comprises a myriad of other biological collections (Liberzon et al., 2015). Tools that perform enrichment analysis may utilize a single source of biological information (such as Reactome and Gene Ontology, which offer enrichment analysis tools but are restricted to their own vocabulary) or perform an integrated analysis of many sources concomitantly such as DAVID (Huang et al., 2007) and Panther (Mi et al., 2013), with the latter tools having the advantage of extracting complementary biological information available at different data sources. It is more important, however, that the utilized underlying data source be current and updated because tools based on outdated annotations can profoundly impact the results of enrichment analysis by effectively underestimating the functional significance of the gene lists used as inputs (Wadi et al., 2016).

Enrichment analysis strategies can generally be grouped into two main approaches: (1) list-based and (2) rank-based methods. The first relies on a set of biomolecules of interest that can be derived from the list of DEGs (if working with RNA-seq data), proteins (if working with proteomics), or compounds, if the obtained data are from metabolomics experiments. To calculate the significance of the enrichment, these tools usually rely on statistical methods based on distributions, such as χ^2^ (chi-squared), hypergeometric, and binomial, and evaluate whether there is an overrepresentation of biomolecules in the corresponding annotations from the data sources (such as genes in a pathway) that could be deemed statistically significant, usually after correcting for multiple hypothesis tests. One of the most used tools in this class is DAVID, which registers over 15,900 citations (Huang da et al., 2009b). A drawback of these approaches is the creation of the gene list itself, as different thresholds (such as those previously indicated for DEG identification) can be used, thus leading to gene lists of variable reliability. Additionally, genes with small expression changes, but having important biological roles, will probably not be included in such lists. Rank-based methods attempt to overcome these limitations by using the complete list of biomolecules as input when performing enrichment analysis, and the list is ranked using an appropriate metric, such as the elements on the top (or bottom) as more biologically important. Kolmogorov-Smirnov-like statistics can then be applied to calculate enrichment significance. In the case of *omics* studies (such as RNA-seq or quantitative proteomics), an appropriate metric could be, e.g., FC-values ordered in a decreasing manner, where the extremes represent biomolecules that are upregulated or downregulated (at the bottom) in a comparison of interest. Alternative metrics could be used for other data types, such as *p*-values and abundance. The Gene Set Enrichment Analysis tool (Subramanian et al., 2005) is among the most popular software for performing this class of analysis. Similar to many enrichment analysis tools, including DAVID, it was originally developed for use with microarrays, but its application to RNA-seq data is also possible. In particular, many tools that were previously restricted to use in microarray data, such as ROAST (Wu et al., 2010), can now also be employed with RNA-seq data using transformation strategies such as the previously described *voom/limma* pipeline (Law et al., 2014), so count-based data can be more closely related to those of microarrays.

Table 1 provides a non-exhaustive list of some of the tools available to perform enrichment analysis fulfilling two criteria: (1) they are currently maintained, and (2) the database annotations on which they rely are updated (at most annually). However, as this field has grown substantially with the advent of high-throughput technologies, a multitude of tools have, in parallel, become available for performing these tasks, and we also refer the reader to specific reviews for a more comprehensive assessment, such as those studies from Huang da et al. (2009b), García-Campos et al. (2015), and Felgueiras et al. (2018), as well as Huang da et al. (2009a) in the Table 1. By focusing on 11 reports in the leishmaniasis community that used RNA-seq data, the use of the *voom/limma* and edgeR's pipeline for the identification of DEGs is indicative that somewhat “standard” tools are in use (Table 2). In particular, *voom/limma* allows microarray-like analysis, and its wide use is probably reminiscent of the extensive application of microarrays by the community, as exemplified by the recent parasite-focused review by Alonso et al. (2018). For enrichment analysis, however, only GSEA appears consistently among studies that performed such analyses, which may be a reflection of the multiplicity of tools available for this purpose. Thus, no clear picture emerges. Three of the 11 studies restricted their analysis to that of the DEG list (Table 2). In summary, the contextualization of lists of interesting biomolecules or pre-ranked sets thereof into the pathway and other cellular processes facilitate the interpretation of results derived from high-throughput data and should be used as complementary approaches to address the biological questions underlying *omics* experiments, thus allowing broader analyses.

**Table 1:** Computational tools for performing functional enrichment analysis using *omics* datasets.

**Tool**	**Year^a^**	**Description and last update^b^**	**No. of citations^c^**	**Type of** ***omics***		**References/URL^d^**
				**RNA**	**Protein**	
DAVID	2003	Free webserver that performs enrichment analysis using various databases (including Biocarta, KEGG, Reactome, GO) based on a **modified Fisher's exact test**. Last update: 2018	15,954	√	√	Huang da et al., 2009a http://david.ncifcrf.gov/
GSEA	2005	Free multi-platform software. Performs rank-based enrichment using annotated gene sets from MSigDB or custom annotations. Calculates an enrichment score based on **weighted Kolmogorov-Smirnov-like statistics**. Last update (MSigDB): 2017	13,892	√	√	Subramanian et al., 2005
Ingenuity Pathway Analysis (IPA)	2004^*^	A paid alternative that combines various analyses tools including functional enrichment (of diseases and biological functions), that is performed based on **Fisher's exact test** using a manually curated ontology and a continuously updated knowledgebase	1,767^$^	√	√	http://www.ingenuity.com
Panther	2003	Allows the performance of **binomial** and **Fisher's exact test** using information from GO, Panther pathways and Reactome	1,732	√	√	Thomas et al., 2003; Mi et al., 2013 http://pantherdb.org
ClueGO	2009	A plugin for Cytoscape that performs enrichment analysis using Gene Ontology, Reactome, and KEGG, also creating network-based visualizations of gene functions. Supports many organisms, and others can be added upon request. Performs enrichment analysis based on the **hypergeometric distribution**. The most recent database annotations can be retrieved automatically	1,338	√	√	Bindea et al., 2009 http://apps.cytoscape.org/apps/cluego
WebGestalt	2005	Free webserver supporting 12 model organisms including human and mouse, and performs both list- (**Fisher's exact test**) and rank-based enrichment of various databases including GO, KEGG, Reactome, Panther, and WikiPathways. Last update: 2017	1,265	√	√	Zhang et al., 2005; Wang et al., 2017 http://www.webgestalt.org
Reactome	2005	Offers a module for enrichment analysis based on a **hypergeometric test** using curated information from the Reactome Knowledgebase	1,124	√	√	Joshi-Tope et al., 2005; Fabregat et al., 2018; http://www.reactome.org
Enrichr	2013	Free webserver that performs enrichment analysis of >40 databases taking as input a list of mammalian genes. Allows various types of visualizations and programmatic access via API. Employs a **modified Fisher's exact test** to perform enrichment analysis	736	√	√	Chen et al., 2013; Kuleshov et al., 2016 http://amp.pharm.mssm.edu/Enrichr
g:Profiler	2007	Free webserver supporting >200 organisms and performing both list- and rank-based enrichment (**hypergeometric distribution-based)** of various databases including GO, KEGG, Reactome, BioGRID (protein-protein interaction), OMIM, TRANSFAC (regulatory), and Human Protein Atlas. Updated quarterly following Ensembl's releases	447	√	√	Reimand et al., 2007 https://biit.cs.ut.ee/gprofiler/
GAGE	2009	A methodological framework that uses a **two-sample** ***t*****-test** to test whether a specific gene-set is enriched relative to a background set	379	√	√	Luo et al., 2009
ConsensusPathDB	2009	Free webserver integrating information from 32 human-related biological databases and allowing enrichment analysis using a **hypergeometric distribution**. Supports only human identifiers (from UniProt, HGNC, Ensembl, Entrez or RefSeq). Last update: 2018 for most databases	240	√	√	Kamburov et al., 2009
ROAST	2010	R function within the *limma* package that performs individual gene set testing based on **multivariate regression**. The user should select pathways of interest based on *a priori* knowledge. RNA-seq data should be processed using the *voom/limma* pipeline to use the package	208	√		Wu et al., 2010

a *Year of original publication*.

b *Date of last update relevant only to tools that rely on embedded or external databases*.

c *Number of citations of the original publication retrieved from Google Scholar, current as of May 2018*.

d *If more than one, the original work and the most recent update are cited*.

$ *Based on PubMed all-time search using “Ingenuity Pathway Analysis” as a query*.

* *Based on PubMed searches for the first usage of the tool published in the literature*.

**Table 2:** Statistical and bioinformatics analyses performed in published articles in the leishmaniasis field that employed RNA-seq and proteomics techniques.

**Authors**	**Year**	**DOI**	**Statistical methods**	**Enrichment analysis methods**	**Network-based method**
**RNA-seq**					
Alcolea et al., 2018	2018	10.1016/j.parint.2018.03.008	Geneious	–	–
Osman et al., 2017	2017	10.1371/journal.pntd.0005527	edgeR	Ingenuity Pathway Analysis, GSEA	–
Masoudzadeh et al., 2017	2017	10.1016/j.actatropica.2017.08.016	edgeR	Gene Ontology website, GSEA	–
Aoki et al., 2017	2017	10.1371/journal.pntd.0006026	*t-*test on FPKM-values estimated by Cufflinks	Performed list-based enrichment analysis using KEGG as database without specifying tool.	–
Iantorno et al., 2017	2017	10.1128/mBio.01393-17	edgeR	–	–
Cuypers et al., 2017	2017	10.1038/s41598-017-03987-0	DESeq2	BiNGO, GSEA	–
Fernandes et al., 2016	2016	10.1128/mBio.00027-16	Voom/limma	ConsensusPathDB, Goseq	–
Christensen et al., 2016	2016	10.1371/journal.pntd.0004992	Voom/limma	GSEA	WGCNA
Dillon et al., 2015	2015	10.1093/nar/gkv656	Voom/limma	ConsensusPathDB, Goseq	–
Willis et al., 2014	2014	10.4049/jimmunol.1303216	Voom/limma	–	–
Maretti-Mira et al., 2012	2012	10.1371/journal.pntd.0001816	CuffDiff	Ingenuity Pathway Analysis	–
**PROTEOMICS**					
Menezes et al., 2013	2013	10.1016/j.micinf.2013.04.005	Sequest algorithm within Bioworks software	Ingenuity Pathway Analysis	Ingenuity Pathway Analysis
Singh et al., 2015	2015	10.1128/IAI.02833-14	ProteinPilot	Gene Ontology	–
Goldman-Pinkovich et al., 2016	2016	10.1371/journal.ppat.1005494	Proteome Discoverer; MaxQuant	–	–

–, *did not perform*.

### Network-based analyses offer a more global view of *omics-*derived data

While enrichment-based methods allow one to obtain a wider view of high-throughput *omics* experiments compared to examining a list of individual biomolecules, a complementary strategy consists of constructing networks of biomolecules. A well-known facet of biological systems is that the different elements (genes, transcripts, and proteins) and scales (genomic, transcriptomic, proteomic, and regulatory) that form these systems are intrinsically connected, such that single pathways or cellular processes seldom occur in isolation in a cell. Instead, the different cellular programs perform in a coordinated manner to achieve their biological functions. This behavior is amenable to modeling using a network-based framework. While there are various ways of applying network-based techniques, in this review, we focus on the construction of correlation networks and module detection approaches, but some methods to infer regulatory patterns are also described.

Correlation networks are being increasingly used to describe correlational patterns in *omics* datasets, and the elements (or nodes) that form these networks can be genes, proteins, or metabolites. In simple correlation networks, an interaction (or edge) between any two nodes is established when the value of their correlation, which can be obtained using Pearson's *r* or Spearman's ρ, passes a given threshold. A number of biological questions have been approached using this framework, and some applications in the context of leishmaniasis include the study of distinct states of infection with *Leishmania infantum* (Gardinassi et al., 2016) and the evaluation of the host-parasite interplay in localized cutaneous leishmaniasis caused by *L. braziliensis* (Christensen et al., 2016). Both studies used expression data as input to construct weighted gene-gene correlation networks, a particular case of a correlation network in which the edges have associated weights and no strict conditional on the correlation values is set, which characterizes a soft-thresholding approach. This method is referred to as weighted gene correlation network analysis (WGCNA) (Langfelder and Horvath, 2008) and has been used to search for biomarkers of psoriasis (Sundarrajan and Arumugam, 2016), various cancer types (Li et al., 2017; Xia et al., 2018; Yuan et al., 2018), as well as other complex, multifactorial conditions such as coronary heart disease (Huan et al., 2013).

Simple correlation networks are constructed by applying a hard-thresholding approach (i.e., reject correlations below a fixed threshold), a strategy that may lead to a loss of information because correlations that fall even slightly below the threshold will be discarded. Defining such limits can also be overly arbitrary and dataset-specific. In contrast, correlation networks constructed using WGCNA mitigate these issues by applying a mathematical transformation to the correlation values, yielding a weighted network where the edge strengths are bounded by the transformed correlation values. The algorithm begins by first obtaining a correlation matrix from the input, usually expression data, but other *omics* data types can also be used. For expression data, correlation is used as a proxy for co-expression, which relates biologically to functional coupling (for instance, a group of co-expressed transcripts probably code for proteins participating in a common process) or regulatory aspects (such as activation of a transcription factor leading to increased expression of the regulated gene). The choice of correlation metric for constructing these networks has been a subject of investigation (Kumari et al., 2012; de Siqueira Santos et al., 2014), and although traditional metrics can be used, the biweight midcorrelation is recommended by the authors of WGCNA as a more robust alternative against outliers in the data (Langfelder and Horvath, 2012). Once all pairwise correlations are calculated, the correlation matrix is transformed into an adjacency matrix using a power function of the form *f*(*x*) = *x*^β^, where *x* represents elements in the correlation matrix, and a value of β ≥ 1 (called the soft-thresholding parameter) is chosen by the user such that the resulting correlation network adheres to a scale-free property while maintaining high connectivity (Langfelder and Horvath, 2008). Because this can lead to a range of valid β-values, an automated selection method has been proposed in the recently published CEMiTool pipeline (Russo et al., 2018). With the correlation network at hand, the next step involves detecting modules of co-expressed genes, which can be performed using hierarchical clustering per default in WGCNA or using hybrid approaches such as an additional K-means clustering step, which has been reported to improve the quality of the disclosed clusters (Botía et al., 2017). Once the modules of correlated bioelements are identified, several downstream analyses can be performed, including functional enrichment, a strategy coupled to a “guilty-by-association” paradigm that can lead to identification of novel gene functions (e.g., genes previously unrelated to a cellular pathway belonging to a module enriched for genes that belong to said pathway). Within a module, the pinpointing of “hub” genes, such as those with more connections, enables further stratification of genes that compose each module. It is also possible to calculate the module eigengene, a metric that summarizes the gene expression/abundance profiles in a module, which is defined by its first principal component (Langfelder and Horvath, 2008). The module eigengene can be correlated to trait data, such as clinical phenotypes and other associated variables in an experiment, and this module eigengene-phenotype association facilitates biomarker identification (Cui et al., 2015; Liu et al., 2015). While WGCNA is not the only tool available to create networks, its simplicity of use and biologically sound results may explain its broad acceptance, as measured by its high number of citations (Table 3). Alternative approaches are shown in Table 3, with some being independent of network inference, as exemplified by coseq (Rau and Maugisrabusseau, 2017), while others are geared toward the elucidation of regulatory interactions, such as ARACNe (Margolin et al., 2006), GENIE (Huynh-Thu et al., 2010), and the commercial alternative IPA (Krämer et al., 2014). It is important to stress that the development of computational tools for biological data analysis is a fast-moving and continuously evolving research field, and while we focused on specific tools and databases that we deemed appropriate and current, alternative solutions (either commercial or open-source) are probably available for performing many of the tasks referred here.

**Table 3:** Computational tools for inferring co-expression and regulatory patterns in *omics* datasets.

**Tool**	**Year^a^**	**Description**	**No. of citations^b^**	**References^c^**
WGCNA	2008	R package for constructing weighted gene correlation networks and module detection using hierarchical clustering	2,721	Langfelder and Horvath, 2008
ARACNe	2006	R package that allows the inference of direct regulatory relationships between transcriptional regulators and target genes based on an information-theoretic approach	1,767	Margolin et al., 2006; Lachmann et al., 2016
Ingenuity Pathway Analysis (IPA)	2014	Paid alternative with modules for “upstream regulator analysis,” “mechanistic networks,” “causal network analysis,” and “downstream effects analysis.” Input can be expression, proteins, metabolites	668	Krämer et al., 2014
GENIE3	2010	R package that uses an ensemble of decision trees (random forest) to perform regression analysis, predicting the expression pattern of one of the target genes from the expression patterns of all other genes	398	Huynh-Thu et al., 2010
coseq	2017	R package that fits Gaussian mixture models for co-expression analysis and cluster detection. A predefined number of clusters (K) should be set *a priori*	4	Rau and Maugisrabusseau, 2017
CEMiTool	2018	R package that automates the module discovery process, selecting the optimal parameters for each input dataset and constructing co-expression networks (based on WGCNA), performs enrichment analysis (using a hypergeometric distribution) and creates high-quality plots and reports	1	Russo et al., 2018

a *Year of original publication*.

b *Number of citations of the original publication retrieved from PubMed*.

c *If more than one, the original work and the most recent update are cited*.

**Figure 1:**
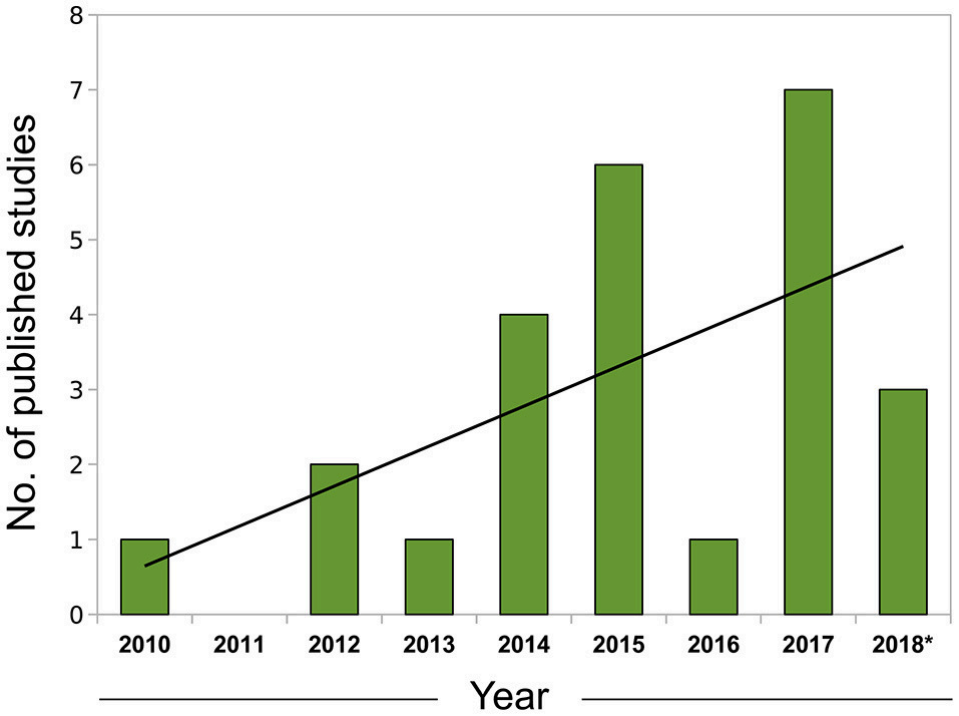
Growth of leishmaniasis studies using RNA-seq indexed in PubMed (2010–2018). Search conducted in May, 2018 using the PubMed query builder with the following phrase: “leishmania*” AND (rna-seq OR rnaseq)—restricted to the Abstract or Title of papers. A linear trend line (black) was fitted to the data. *Data for 2018 comprehends Jan. to Apr.

In summary, correlative approaches offer an alternative way of examining *omics* datasets in a completely data-driven fashion. Although we have focused mostly on expression data to exemplify the use of this technique, correlation networks are agnostic to the data type and can be constructed using any biomolecule with interactions that are amenable to modeling using a systems framework, including proteins (e.g., Zhang et al., 2016) and metabolites (e.g., Dileo et al., 2011) and the specificities involved in the adaptation for each data type were the subject of a recent investigation (Pei et al., 2017). The coupling of network creation and module detection with enrichment methods permits researchers to conduct more integrative analyses and extract biological insights in a much richer way than in traditional, single-gene based approaches. With the current trend of expanding the adoption of *omics*, particularly RNA-seq data, by the leishmaniasis scientific community (Figure 1), the knowledge and application of these more advanced computational techniques will be of utmost importance for progress in the field.

## Transcriptomics contribution to understanding the host response to *Leishmania* infection

Thus far, we have presented some of the analytical hurdles involved in the analysis of *omics* datasets. In the following sections, we focus on how the use of high-throughput approaches allowed an improved comprehension of *Leishmania* infection and host interplay.

Several studies have explored the advantages of transcriptome profiling using RNA-seq vs. other techniques to identify, analyze, and quantify transcriptomes from a variety of eukaryotic organisms. Most importantly, in comparison to other transcriptome sequencing techniques, RNA-seq offers improvements in terms of quality and precision regarding the level of transcripts and their isoform measures (Wang et al., 2009; Oshlack et al., 2010). A recent study highlighted the importance of RNA-seq as a tool to reveal gene expression at different stages of protozoan parasite development and to identify parasite genes modulated by vertebrate and invertebrate hosts via the simultaneous sequencing of parasite and host cell transcripts (Patino and Ramírez, 2017). A series of comprehensive studies attempted to investigate host cell signatures in response to *Leishmania* spp. infection by identifying not only DEGs but also modulated pathways using enrichment analysis, as discussed in section Enrichment Analyses Allow a Contextualization of Altered Biological Processes in High-Throughput Data of this review. These studies have greatly expanded our knowledge regarding the virulence mechanisms of these parasites and their interactions with hosts (Guerfali et al., 2008; Dillon et al., 2015; Novais et al., 2015; Christensen et al., 2016; Fernandes et al., 2016). Although beyond the scope of the present review, we must acknowledge some recent studies that aimed to investigate the gene-wide transcriptional profiles of cutaneous lesions from patients infected with *Leishmania braziliensis* (Maretti-Mira et al., 2012; Novais et al., 2015; Christensen et al., 2016). One of these studies comparatively evaluated gene expression in lesions from patients who developed mucosal leishmaniasis and those who did not (Maretti-Mira et al., 2012). Another investigated gene expression in *L. braziliensis*-infected cutaneous lesions in comparison to normal skin (Novais et al., 2015), and a third report simultaneously analyzed the transcriptomic profiles of *L. braziliensis* amastigotes derived from skin lesions in *L. braziliensis*-infected patients and lesion skin samples by comparing profiles at early and late stages of disease and comparing lesions lacking detectable parasite transcripts and lesions with parasite transcripts that were readily detected, and used weighted gene-gene networks to globally assess the human host gene expression (Christensen et al., 2016). In addition, although comprehensive studies using microarray technology have contributed to the understanding of the host gene expression profile in response to parasites that cause visceral leishmaniasis (Gardinassi et al., 2016), we were unable to identify any RNA-seq studies analyzing the response to these parasite species. Another aspect that should be taken into consideration is metabolic changes induced in host cells by *Leishmania* parasites. While we recognize that metabolomic analysis represents an important aspect that has recently been explored in the field of leishmaniasis (Armitage et al., 2018; Cuypers et al., 2018), which certainly contributes to the understanding of disease, the results from these studies fall outside the scope of the present study. The present review instead focuses on global transcriptome analysis of macrophages in response to infection, which has been poorly investigated using RNA-seq technology (Dillon et al., 2015; Fernandes et al., 2016).

### Transcriptomic analysis allowing the opportunity to identify possible biomarkers in *Leishmania*-infected macrophages

Recent studies that analyzed gene expression profile in host cells have demonstrated that early stages [4 hours post-infection (hpi)], as opposed to later time points after infection (24, 48, and 72 h), seem to be ideal for the identification of DEGs or specifically modulated pathways in mouse or human macrophages (Dillon et al., 2015; Fernandes et al., 2016). This notion is supported by a well-designed protocol that used not only uninfected human macrophages as controls but also cells that engulfed inert particles to comprehensively distinguish genetic expression induced by phagocytosis from that arising upon infection, which has been shown to be indistinguishable at later infection times (Fernandes et al., 2016). Therefore, this study was able to capture the unique response of macrophages to each of the two *Leishmania* species investigated, *Leishmania major* and *Leishmania amazonensis*, which can potentially cause different clinical manifestations, by excluding the effect on human macrophages to inert particles. Interestingly, using principal component analysis (PCA), both human macrophages and cells incubated with latex beads for 4 hpi were shown to be clustered together, indicating that macrophages in culture can undergo phagocytosis without disturbing their steady-state transcriptome. As previously described (Vieira et al., 2002; Lee et al., 2007), phagocytosis triggers the activation of a local cascade of events that results in a cytoskeletal imbalance and formation of the phagocytic cup. By contrast, infected human macrophages seem to activate a unique transcriptional profile in response to *Leishmania* parasites at 4 hpi, regardless of species, since *L. major*- and *L. amazonensis*-infected cells have been shown to cluster together (Fernandes et al., 2016). This approach allowed the identification of specific genes that are expressed in response to infection, including potential macrophage biomarkers.

An evaluation of changes in the transcriptomic response to *Leishmania* infection over time revealed that murine and human macrophage responses to infection at early stages of infection vary significantly from those observed at later timepoints, by demonstrating that the number of DEGs, in comparison to uninfected macrophages, is higher in *L. major* and *L. amazonensis*-infected human macrophages at 4 hpi, with decreasing quantities observed at later time points. By contrast, infected human macrophages activate a similar transcriptomic response to uninfected macrophages that internalized inert particles at 24 hpi. In consonance with this finding, these two populations of macrophages, as well as uninfected control macrophages, all clustered together at 48 and 72 hpi (Fernandes et al., 2016). Evaluation of the phagocytotic effect on gene transcription demonstrated a lack of response in bead-containing macrophages at 4 hpi, with no DEGs observed between these macrophages and uninfected cells, although highly pronounced differences were detected at later time points. These findings indicate that, in contrast to the response exhibited by uninfected macrophages and macrophages that internalized the latex beads, *Leishmania* triggers a unique transcriptomic response shortly after phagocytosis, with reduced communication between the parasite and host cell at later stages of infection (Fernandes et al., 2016).

Similar to what was observed in human macrophages, in comparison to uninfected cells, *L. major*-resistant C57BL/6 macrophages were also shown to differentially modulate the variable numbers and types of genes at 4 hpi vs. later timepoints. At all tested time points, only 47 genes were up- or downregulated, which did not seem to be functionally related, except for the heavy metal transporters metallothionein 1 and 2 (Dillon et al., 2015). In *L. major*- and *L. amazonensis*-infected human macrophages, metallothionein 1 family members were also found to be some of the most upregulated (up to a 136-fold increase during *L. major* infection and a 196-fold increase in response to *L. amazonensis* infection, both compared with uninfected cells). These potential biomarkers are proteins that have previously been associated with an immunomodulatory response (Lynes et al., 1993) and are known to be activated by certain stimuli, such as exposure to reactive oxygen species (Ghoshal and Jacob, 2001), which has been confirmed to influence the host response to *Listeria* spp. (Emeny et al., 2015). Metallothioneins have also been found to be highly upregulated in macrophages infected with *Leishmania* (Chaussabel et al., 2003; Ettinger and Wilson, 2008) and have also been associated with resistance to treatment with antimonial drugs (Gómez et al., 2014). Despite this insight, the actual role played by these proteins in the establishment of *Leishmania* infection warrants further investigation.

Although *L. major* and *L. amazonensis* differ in several aspects of interaction with host cells (Kaye and Scott, 2011; Real et al., 2014), they surprisingly trigger a quite similar global transcriptomic response in human macrophages, with only four genes known to be differentially expressed at 4 hpi, compared to none at subsequent time points. This finding seems to indicate that human macrophages possess a nominal ability to distinguish between *L. major* and *L. amazonensis* at the transcriptional level, despite differences in several aspects of the clinical presentation of tegumentary leishmaniasis caused by these parasite species, as well as host immune response (Fernandes et al., 2016). This finding indicates that, in the search for novel biomarkers, it is likely that only those that would be similarly detected in macrophages, regardless of the parasite species that causes disease, will be identified. Notably, among the few DEGs identified between *L. major*- and *L. amazonensis*-infected macrophages, the authors reported that two were involved in the essential mechanisms of parasite establishment inside host cells: synaptotagmin family members 2 and 8 (SYT2 and SYT8), which are membrane proteins implicated in the regulation of vesicle docking and fusion in exocytosis (Baram et al., 1999; Arango Duque et al., 2013) and phagocytosis (Czibener et al., 2006; Vinet et al., 2008; Arango Duque et al., 2013). Although other synaptotagmin family members, SYT5 and SYT11, have been implicated in *Leishmania* infection, the roles played by SYT2 and SYT8 require further investigation. It has been proposed that the higher expression levels of SYT2 and SYT8 observed during *L. major* infection may be linked to differences in *L. major*-induced vacuole maintenance throughout the course of infection in terms of how the parasites divide within these compartments, i.e., the maintenance of a single parasite in one vacuole upon division, in contrast to *L. amazonensis*, which inhabits large parasitophorous vacuoles that potentially require more fusion (Veras et al., 1994, 1996) instead of fission events (Fernandes et al., 2016). Synaptotagmins are also involved in the regulation of SNARE activity by influencing membrane fusion via a Ca^2^-dependent mechanism (Tucker and Chapman, 2002; Andrews and Chakrabarti, 2005; Südhof and Rothman, 2009).

In comparison to uninfected cells, C57BL/6 macrophages infected with *L. major* upregulated two genes (*Bnip3* and *Bcl2a1b*) related to the Bcl2 inhibitor of apoptosis, which is associated with inhibiting macrophages from resisting cell death (Dillon et al., 2015). How this finding is associated with a resistance profile in this murine model of leishmaniasis seems unclear. Previously, it was demonstrated that murine bone marrow-derived macrophages infected with *L. major* exhibited reduced programmed cell death when induced by stimuli, such as the deprivation of growth factors or treatment with staurosporine. Interestingly, this preventive effect was detected in both macrophages from *L. major*-susceptible BALB/c and *L. major*-resistant C57BL/6 mice, suggesting that the observed reduction in programmed cell death might be a parasite-triggered process that is seemingly independent of host genetic background and is unrelated to resistance and susceptibility to infection (Akarid et al., 2004).

### Integrative bioinformatics analyses offer a comprehensive view of sets of possible biomarkers in *Leishmania*-infected macrophages

As discussed initially, the identification of pathways using database resources aids in a more complete understanding of the global response of host cells and tissues to a specific microorganism. A comprehensive analysis of these pathways using e.g., KEGG could help identify those genes that represent potential targets for disease intervention. In the C57BL/6 murine infection model involving *L. major*, the most highly modulated macrophage gene expression was related to the immune response, which is consistent with the resistance observed in these mice. Some of the upregulated genes that clustered together under KEGG analysis were *Tnf*, *Hif-1, NF-kappa-B, Jak-Stat, PI3K-Akt*, and *Mapk*, which are involved in cytokine-cytokine receptor interactions, arginine and proline metabolism, glycolysis and signaling pathways (Fernandez-Figueroa et al., 2016). Transcripts for inflammatory cytokines and their receptors were also found to be upregulated in *L. major*-infected mouse macrophages, including *Il1, Il6, Tnf*, *Il1rap, Il18r1*, and *Nos2*. In addition, KEGG enrichment analysis showed that murine macrophages infected with *L. major* expressed genes involved in the anti-inflammatory response, including *Il11r, Il1rn, Il10, Socs3, Fos-induced growth factor* (*Figf*), *hemoxygenase1* (*Hmox1*), *epithelium growth factor receptor* (*Egfr*), *vascular endothelial growth factor* (*Vegf*), *colony-stimulating factor 1* (*Csf1*), and *colony-stimulating factor 3* (*Csf3*) (Weis et al., 2009; Luz et al., 2012; Canavese et al., 2015). Accordingly, the responses observed in human macrophages infected with *L. major* at 4 hpi were similar to those of murine macrophages, resulting in the upregulation of genes encoding inflammatory cytokines, including *Il1* and *Il6*, and the upregulation of immune regulatory genes, including *prostaglandin endoperoxide synthase 2* (*Ptgs2*), *Csf1* and *colony-stimulating factors 2* (*Csf2*), and *superoxide dismutase 2* (S*od2*). This finding suggests that *L. major*-infected macrophages probably evolved the ability to inhibit a deleterious innate inflammatory immune response (Fleming et al., 2015); alternatively, this anti-inflammatory response could be a consequence of the effort by host macrophages to control parasite infection (Dillon et al., 2015). Consistent with these findings, in the sera of patients during the active phase of visceral leishmaniasis it was detected a significant increase in inflammatory mediators including LTB4, RvD1, PGF2α (PGF2α), IL-1β, IL-6, IL-8, IL-10, IL-12p70, and TNF-α, and a decreased level of TGF-β1 (Araújo-Santos et al., 2017).

Enrichment analysis involving C57BL/6 macrophages infected with *L. major*, conducted at 4 and 24 hpi, identified activation of the glycolysis/gluconeogenesis pathway, which contains genes that encode glycolytic enzymes, such as phosphoglycerate kinase, hexokinases, enolase, lactate dehydrogenase A, and glyceraldehyde-3-phosphate dehydrogenase. This finding seems to indicate that the glycolysis pathway represents a metabolic response arising in macrophages due to *L. major* infection, which, upon toll-like receptor ligation, likely results in the stimulation of an inflammatory response capable of triggering anaerobic glycolysis (Tannahill et al., 2013). Whether this metabolic response to *Leishmania* spp infection is typical of host macrophages or whether it is due to host resistance to infection warrants further study.

Few pathways have been found to be downregulated in the *L. major* murine infection model. At 4 hpi, downregulation of the lipid metabolism and biogenesis pathways was observed. In addition, in the “Fc gamma R-mediated phagocytosis” KEGG pathway, receptors and signaling molecules involved in the process of phagocytosis were downmodulated at 4 hpi. Previously, it has been demonstrated that macrophages are more permissive to IgG-opsonized-*Leishmania* phagocytosed by the Fc gamma receptor (Mosser, 1994). It is possible that the observed resistance to *L. major* could be related to a possible reduction in the uptake of *L. major* by C57BL/6 macrophages secondary to the downregulation of this pathway. However, the mechanism underlying this effect in this murine model and resistance to *L. major* by C57BL/6 macrophages in general requires further investigation.

Fernandes et al. (2016) generated transcriptomic data from infected cells and integrated those data with the database from a previous study (Dillon et al., 2015) to define a shared response that characterizes a general mammalian macrophage gene signature in response to *Leishmania* spp. infection. To identify known cellular processes within this signature, KEGG enrichment analysis was used to ascertain which genes were commonly up- or downregulated in infected cells. Most of the pathways identified contained upregulated genes that were related to immune activation and signaling responses. Regarding signaling pathways, KEGG analysis identified genes involved in the pathway of recognition of pathogen associated molecular patterns (PAMPs), e.g., retinoic acid-inducible gene-(RIG)-I-like receptor, nucleotide-binding oligomerization domain-NOD-like receptor, and Toll-like receptor; for the immune system signaling pathways, the detected genes were either related to the cytokine-cytokine receptor interaction pathway, including Fc epsilon RI, Jak-STAT, T cell receptor, NF-kB, mitogen-activated protein kinase (MAPK), TNF, vascular endothelial growth factor (VEGF), ErbB, FoxO, hypoxia-inducible factor 1 (HIF-1), and phosphatidylinositol 3-kinase-Akt [PI3KAkt], or related to the TGF-β signaling pathway. In addition, among the downregulated genes in both murine and human models of infection, KEGG identified pathways related to energy metabolism (glycan and amino acid degradation), lysosome structure and processes and apoptosis. KEGG enrichment analysis identified the FoxO signaling pathway among the genes that were either up- or downregulated, which is implicated in the regulation of cell growth, gluconeogenesis, and adipogenesis. The findings presented in RNA-seq technology raise the possibility of translating these pathways to biomarkers as surrogate endpoints following extensive validation studies.

## Proteomic contribution to understanding the macrophage response to *Leishmania* infection

Different DNA- and RNA-based strategies have been used to provide insights into the host cell response to infection by different pathogens, including *Leishmania*. However, these studies did not provide information regarding translational and post-translational modifications and protein localization, which are essential to understanding gene functions. Thus, studying the proteins encoded by mRNAs is crucial for understanding the biological processes. Therefore, proteomic studies have gained significant relevance with the advancements in large-scale technologies and represent one of the most important tools for biomarker investigation. This approach has provided a wealth of protein expression data on the host response to infection by different pathogens (Chambers et al., 2000; Sundar and Singh, 2018).

Although proteomics is a known powerful tool to identify host cell protein expression (Chambers et al., 2000), only three studies have evaluated the macrophage response to *Leishmania* infection in the past 5 years. A previous work published by our group, using tandem liquid chromatography-mass spectrometry (LC-MS/MS), was the first attempt to employ a large-scale proteomic analysis to identify host cell proteins expressed in response to *Leishmania* infection and, among them, potential macrophage biomarkers that could be related to a susceptibility or resistance profiles (Menezes et al., 2013). Two years after this paper was published, Singh et al. (2015) used a quantitative proteomic approach to study human monocyte-derived macrophage (THP-1) responses to *L. donovani* infection to investigate how the intracellular parasite manipulates the macrophage response. More recently, Goldman-Pinkovich et al. (2016) applied a phosphoproteomic analysis to understand the arginine deprivation response in infected macrophages and the underlying mechanisms.

In the first study, our group used a mouse model that was previously described as being resistant to *L. major* and susceptible to *L. amazonensis*, to identify markers that could be driving different responses of CBA mouse macrophages to *Leishmania* infection. A total of 62 proteins were predominantly expressed in infected macrophages. Of those, 15 proteins were found to be differentially expressed between *L. amazonensis-* and *L. major-*infected macrophages. Thirteen of the 15 proteins exhibited reduced expression in response to *L. amazonensis* infection, but they were upmodulated in *L. major*-infected macrophages; in contrast, two proteins showed increased expression in response to *L. amazonensis* infection. The proteins with higher expression in *L. major*-infected macrophages were as follows: programmed cell death protein 5 (PDCD5), coronin 1B, HIF-1α, cytochrome C oxidase 6B (cox6B), osteoclast-stimulating factor-1 (OSTF1), protein phosphatase 2 (PP2), heterogeneous nuclear ribonucleoprotein F (HNRPF), PYD And CARD domain-containing protein (PYCARD), RAB1, Serpin, ribosomal protein S2 (RPS2), and myosin light chain (Menezes et al., 2013). Networks constructed under the IPA framework revealed that proteins differentially expressed in CBA macrophages form part of biological modules related to cellular development and cellular metabolism, and their different modulation profiles possibly induce distinct macrophage responses, ultimately leading to disease susceptibility or control (Menezes et al., 2013). The upregulation of proteins such as HIF-1α, TRAP1, Serpin, and PYDCARD strongly suggest a modulation of the immune response after *Leishmania* infection. Two of these proteins, Serpin and PYDCARD, were downmodulated in *L. amazonensis-*infected macrophages. Serpin is a protein induced by TNF-α that, together with IL-1β, is involved in the inflammatory cascade (Mishra et al., 2006). The reduced expression of Serpin in *L. amazonensis*-infected macrophages could be associated with a diminished inflammatory response, favoring the intracellular survival of the parasite. Additionally, the PYDCARD adapter protein also induced by TNF-α activates apoptosis via a mechanism that is dependent on NF-κB and caspases (Reed et al., 2003). These results are in accordance with a previous study performed in our laboratory, showing that CBA macrophages control *L. major* infection and express higher levels of TNF-α than *L. amazonensis-*infected macrophages (Gomes et al., 2003), which are susceptible to this parasite (Diefenbach et al., 1998).

Another critical molecule identified in this study as differentially expressed between *L. amazonensis*- and *L. major*-infected macrophages is HIF-1α. The higher levels of this protein in macrophages infected by *L. major* could be associated with higher production of NO and expression of TNF, which are mediators that are known to play a role in HIF-1α regulation (Zhou et al., 2003). Additionally, investigation of the role of HIF-1α in *Leishmania* infection led us to the discovery of 17-AAG, a heat-shock protein-90 (HSP90) inhibitor, as a potential drug against leishmaniasis (Petersen et al., 2012; Santos et al., 2014). HIF-1α, a transcriptional factor that can potentially be modulated by specific drugs, is one of the client proteins of HSP-90, which is a very plentiful molecular chaperone in mammalian cells (Minet et al., 1999). This ATP-dependent chaperone, which is induced during stress responses, is known to play a role in the stabilization, correct folding and assembly of several client proteins, including HIF-1α. HSP90 is also expressed by protozoan parasites, which is crucial to the stabilization of heat-labile proteins inside these microbes. Treatment of *L. amazonensis*- or *L. braziliensis*-infected macrophages with 17-AAG dramatically reduced not only the percentage of infected cells, but also parasite load, in a dose- and time-dependent manner together with decreases in the production of inflammatory cytokines (Petersen et al., 2012; Santos et al., 2014). More recently, we investigated the effect of modulating another identified biomarker using proteomic analysis, the peripheral benzodiazepine receptor (PBR), known as translocator protein (TSPO). We found that this mitochondrial transmembrane protein exhibited a lower relative abundance of peptides in cells infected with *L. amazonensis* in comparison to *L. major* (Menezes et al., 2013). Modulating TSPO with one of its ligand, PK11195, caused the killing of amastigotes *in vitro* at dosages considered non-toxic to macrophages, indicating its potential as antileishmanial (Guedes et al., 2018). In sum, these findings strengthen the potentiality of global analysis of *Leishmania*-infected macrophages for the identification of biomarkers in host cells that probably participate in the pathogenesis of *Leishmania* infection and, subsequently, can function as targets for therapeutic intervention.

The proteomic study described herein also reveals a modulation of host cell metabolism induced by *L. amazonensis*. The results demonstrate that macrophages infected with *L. amazonensis* express higher levels of 6-phosphogluconate dehydrogenase (6PGDH), an enzyme in the pentose phosphate pathway, compared to *L. major*-infected cells (Menezes et al., 2013). The modulation of host cell metabolism induced by *Leishmania* has already been explored (Osorio Y Fortea et al., 2009; Lamour et al., 2012). The modulation of 6PGDH in cancer cells and its effect on cancer treatment are currently being studied (Zheng et al., 2017).

Another recently published study used a quantitative proteomic approach and THP-1-derived macrophages to evaluate the cell host response to *L. donovani* infection (Singh et al., 2015). The authors used the isobaric tag (iTRAQ) method and LC-MS/MS to compare the protein profiles of non-infected and *L. donovani*-infected THP-1 cells, and then performed an extensive analysis for contextualizing their results into ampler biological processes, which facilitated a global interpretation of the altered processes in response to infection. This analytical strategy is beneficial to obtain a comprehensive understanding of the studied phenomenon. The results showed that proteins involved in important metabolic pathways, such as glycolysis and fatty acid oxidation, were upregulated after *L. donovani* infection, suggesting that this parasite modulates host cell metabolism. The expression of proteins involved in gene transcription, RNA splicing [heterogeneous nuclear ribonucleoproteins (hnRNPs)], histones, and DNA repair and replication was also upregulated after *L. donovani* infection. Of note, several proteins identified in this study as differentially expressed between non-infected and *L. donovani*-infected macrophages had not been previously associated with the host cell response to *Leishmania* infection. Another exciting result of this work was the increased expression of the mitochondrial antiviral signaling protein (MAVS) after *Leishmania* infection. This protein is known to activate NF-κB and interferon (IFN) regulatory factors (IRF3 and IRF7), inducing the synthesis of type I interferons (IFN-α and IFN-β), which are essential during antiviral signaling. The silencing of endogenous MAVS expression by RNAi inhibits the activation of NF-κB, IRF3, and IRF7, leading to the blockade of interferon production and favoring viral infection (Yan and Tsai, 1999). These authors suggest that a crosstalk might occur between MAVS and NF-κB and IRF signaling pathways components, which would lead to the production of proinflammatory cytokines and type I IFN (Villa et al., 2003). Based on these findings, MAVS could be an interesting potential marker to investigate because it helps modulate the host inflammatory response to *Leishmania* infection. In addition, the modulation of host cell metabolism could be an interesting approach that could contribute to the control of *Leishmania* infection. Metabolomics combined with proteomic approaches represents one of the most important postgenomic analyses to investigate changes in cell metabolism and identify biomarkers during the course of infection inside macrophages (Singh et al., 2015). Several studies have demonstrated an association between host cell metabolism and response to different pathogens, including *Leishmania* (Lamour et al., 2012; Govinden et al., 2018; Price et al., 2018; Reddy et al., 2018).

The most recent study using a proteomic approach to better understand the host cell response to *Leishmania* infection applied this technology to investigate the signaling pathways involved in the upregulation of expression and activity of different transporters, such as *Leishmania* arginine transporter (LdAAP3), in response to arginine pool reduction in the host cell. To study phosphoproteins involved in the signaling pathway implicated in this response, the authors used a di-methylation tagging technique to investigate changes in the phosphorylation profile of *Leishmania* promastigotes after 5 and 15 min of arginine deprivation. Phosphoproteomic analysis revealed an increased phosphorylation of mitogen-activated protein kinase 2 (MPK2), indicating that this kinase could be involved in the arginine-deprivation response during *Leishmania* infection (Goldman-Pinkovich et al., 2016). Although this work did not investigate a more global cell host response to *Leishmania* infection, the utilized approach could be of great importance to identify potential markers that could be used for the development of new drug treatments and to understand the disease outcome.

Taken together, these few studies show that *Leishmania* parasites modulate the host cell proteome profile, reinforcing the idea that proteomic technology is a powerful technique that should be further explored by researchers to discover its full potential. Proteomics combined with bioinformatics represents a robust approach to investigate the global host response to infection and to identify new potential molecular markers that can control the fate of both host cell and pathogen during infection (Jean Beltran et al., 2017). In addition, further proteomic studies are required to investigate whether proteins that are modulated after *Leishmania* infection can be used as novel biomarkers and targets for the control of *Leishmania* infection.

## Conclusions

Combining the results from transcriptomic and proteomic investigations offers a more comprehensive body of information for the identification of possible biomarkers in *Leishmania* infection. The authors recommend compiling the findings from the studies referenced herein using macrophages, together with those obtained from blood, tissue and other cell types, and also relevant results from similar future studies, to form a complete set of potential biomarkers to aid in global analysis using transcriptomics, proteomics and metabolomics approaches. This data could be then used to identify and subsequently validate specific genes and proteins capable of enhancing the ability of researchers to identify host cell signatures at early time points in the context of leishmaniasis, in an effort to predict disease control or progression, and even the prognostic response to therapy.

## Author contributions

PV was the main responsible for conception and design and also for the formulation of the final version of this article review. PR and JdM made substantial contributions to conception and design, and also participate in drafting the article or revising it critically for important intellectual content.

### Conflict of interest statement

The authors declare that the research was conducted in the absence of any commercial or financial relationships that could be construed as a potential conflict of interest.
